# Personalized risk‐based screening for diabetic retinopathy: A multivariate approach versus the use of stratification rules

**DOI:** 10.1111/dom.13552

**Published:** 2018-10-30

**Authors:** Marta García‐Fiñana, David M. Hughes, Christopher P. Cheyne, Deborah M. Broadbent, Amu Wang, Arnošt Komárek, Irene M. Stratton, Mehrdad Mobayen‐Rahni, Ayesh Alshukri, Jiten P. Vora, Simon P. Harding

**Affiliations:** ^1^ Department of Biostatistics Institute of Translational Medicine, University of Liverpool Liverpool UK; ^2^ Department of Eye and Vision Science Institute of Ageing and Chronic Disease, University of Liverpool Liverpool UK; ^3^ St Paul's Eye Unit Royal Liverpool University Hospital Liverpool UK; ^4^ Department of Probability and Mathematical Statistics Faculty of Mathematics and Physics, Charles University Prague Czech Republic; ^5^ Gloucestershire Retinal Research Group Gloucestershire Hospitals NHS Foundation Trust, Cheltenham General Hospital Cheltenham UK; ^6^ Department of Medical Physics and Clinical Engineering Royal Liverpool University Hospital Liverpool UK; ^7^ Diabetes and Endocrinology Royal Liverpool University Hospital Liverpool UK

**Keywords:** cohort study, diabetic retinopathy, observational study, primary care

## Abstract

**Aims:**

To evaluate our proposed multivariate approach to identify patients who will develop sight‐threatening diabetic retinopathy (STDR) within a 1‐year screen interval, and explore the impact of simple stratification rules on prediction.

**Materials and methods:**

A 7‐year dataset (2009‐2016) from people with diabetes (PWD) was analysed using a novel multivariate longitudinal discriminant approach. Level of diabetic retinopathy, assessed from routine digital screening photographs of both eyes, was jointly modelled using clinical data collected over time. Simple stratification rules based on retinopathy level were also applied and compared with the multivariate discriminant approach.

**Results:**

Data from 13 103 PWD (49 520 screening episodes) were analysed. The multivariate approach accurately predicted whether patients developed STDR or not within 1 year from the time of prediction in 84.0% of patients (95% confidence interval [CI] 80.4‐89.7), compared with 56.7% (95% CI 55.5‐58.0) and 79.7% (95% CI 78.8‐80.6) achieved by the two stratification rules. While the stratification rules detected up to 95.2% (95% CI 92.2‐97.6) of the STDR cases (sensitivity) only 55.6% (95% CI 54.5‐56.7) of patients who did not develop STDR were correctly identified (specificity), compared with 85.4% (95% CI 80.4‐89.7%) and 84.0% (95% CI 80.7‐87.6%), respectively, achieved by the multivariate risk model.

**Conclusions:**

Accurate prediction of progression to STDR in PWD can be achieved using a multivariate risk model whilst also maintaining desirable specificity. While simple stratification rules can achieve good levels of sensitivity, the present study indicates that their lower specificity (high false‐positive rate) would therefore necessitate a greater frequency of eye examinations.

## 1 INTRODUCTION

Early detection and treatment of sight‐threatening diabetic retinopathy (STDR), a stage of diabetic retinopathy (DR) requiring referral to an ophthalmologist, is important to avoid visual impairment in people with diabetes (PWD).^1^, ^2^, ^3^, ^4^, ^5^, ^6^, ^7^ Risk factors for the development and progression of DR have been identified in epidemiological and observational studies.^8^, ^9^, ^10^, ^11^, ^12^, ^13^, ^14^, ^15^, ^16^, ^17^, ^18^, ^19^, ^20^, ^21^ Evidence of the influence of clinical variables in DR progression, such as duration of diabetes, glycated haemoglobin (HbA1c) and type of diabetes, has been widely reported.^3^, ^8^, ^9^, ^19^, ^20^, ^21^, ^22^, ^23^

Given the low annual incidence rate of STDR in the population with diabetes in developed countries (<3%), personalized risk‐based screening should offer a cost‐effective approach to reduce the economic burden on health systems without compromising efficacy.^8^, ^9^, ^24^ Identifying STDR early is important, not just to enable prompt and effective treatment and thus maintenance of vision, but also to allow cost‐effective screening intervals tailored to patients' needs. The potential benefit of a personalized screening approach (where patients at higher risk are screened more often than those at low risk) depends on the accuracy and validity of the predictive model used and on the associated costs. Data collected from a patient over time (longitudinal data) that capture changes in clinical markers could be used to improve the accuracy of a predictive model. In the present study, the individual trajectories of the clinical profiles in PWD were used to develop and validate a predictive model for STDR.

We jointly modelled demographic and clinical data to characterize the baseline level of retinopathy and changes in level of retinopathy over time. We applied a risk‐based longitudinal multivariate approach that enables the identification of patients who will develop STDR within 1 year from the time of prediction. In the United Kingdom and elsewhere, there is a current debate as to whether a simple rule based on the patient's level of retinopathy alone or alternative multivariate clinical models should be used to determine risk‐based screening intervals for STDR.^21^, ^22^, ^23^, ^24^, ^25^, ^26^, ^27^ Risk stratification for development of STDR based on just the results of two screening episodes has been proposed by Stratton et al.^25^ They observed that the annual rate of progression to STDR was 0.7% for patients with no DR at two consecutive annual digital photographic screenings, 1.9% for patients with no DR in either eye at first screening but mild non‐proliferative DR (NPDR)/background DR (BDR) in just one eye at second screening, and 11% for patients with mild NPDR/BDR in both eyes at both screenings. In the present study we compare the overall accuracy of our multivariate model with two simple risk stratification rules, including the rule by Stratton et al., which has been agreed by the UK National Screening Committee to be introduced in England within the next few years.^26^

## 2 MATERIAL AND METHODS

### 2.1 Study participants and design

Data from 13 103 PWD, registered with a general (family) practice in Liverpool, were included in our model. Demographic and systemic risk factor data from primary care systems (EMISweb; EMIS Health Ltd, a company based in Leeds which supplies electronic patient record systems and software used in general practice in England) and level of retinopathy obtained from the Liverpool Diabetic Eye Screening Programme (49 520 screening episodes, from 2009 to 2016, with a median follow‐up of 6 years; OptoMize, EMIS Health Ltd) were linked in a purpose‐built data warehouse. Patients were offered annual screening for DR according to national recommendations.^28^ When patients did not attend their first appointment for screening they were offered a second appointment (usually within 6 weeks of the first appointment). At screening appointments, patients had at least two 45° digital retinal photographs taken per eye, according to national guidelines, which were graded by accredited technicians to assess the level of DR.

A data‐sharing agreement allowed access to general practice data via the Liverpool Clinical Commissioning Group. Practices were approached between 2013 and 2016 and all 92 within the Liverpool area agreed to participate. Patient consent was sought via an opt‐out approach approved by the local research ethics committee (13/NW/0196). Data from PWD who had opted out of the study (7.3%) were not considered for the analyses. Patients with only one clinic visit (8.5%) or with no recorded clinical visit within a time window of 18 months before the final visit (8.7%) were also excluded from the analysis.

### 2.2 Study variables

Available demographic and systemic risk factor data included age, sex, ethnicity, recorded diagnosis of diabetes (time and type), attendance for screening, HbA1c, diastolic blood pressure (DBP) and systolic blood pressure (SBP), total cholesterol and eGFR recorded over time during follow‐up. The following disease states were considered for each eye: (a) no DR detected, (b) non‐referable DR (mild NPDR/BDR) and (c) STDR, defined as moderate/severe pre‐proliferative DR or proliferative DR and/or maculopathy, that is, with any of the following features: multiple blot haemorrhages; venous beading; intraretinal microvascular abnormalities; new vessels; preretinal/vitreous haemorrhage; fibrovascular proliferation; exudates within one disc diameter (1500 μm) of the foveal centre; group of exudates within the macula >0.5 disc area in size; or retinal thickening within 1 disc diameter of the foveal centre.

Patients with STDR at the start of the prediction period were excluded from the analysis. For the purposes of the present analysis, the values of the time‐dependent clinical variables closest to the time of the screen episodes (ie, annual screening episodes) were used. A complete case analysis, similar to that used by Scanlon et al.,^9^ was followed, and screening visits for which model covariates were not available were excluded from the final model.

### 2.3 Model development and statistical analysis

We have recently developed a multivariate discriminant approach, which can be used to predict the future status of a patient using their clinical history.^29^, ^30^ In the present study, we applied this statistical approach to estimate the risk that a patient would develop STDR in either/both eyes within a 1‐year period, and this was achieved by using the demographic and longitudinal primary care data to jointly model the changes in level of retinopathy over time for both eyes (Figure S1). Our approach is based on the following rationale: two longitudinal models are generated using part of the dataset (training dataset), one for each of two possible prognostic groups (patients who develop STDR and patients who do not develop STDR within 1 year). These two models focus on modelling the progression from no DR to mild NPDR/BDR, making use of the biochemical and demographic records of the patients. The status of a new patient is then predicted depending on which of the two models the new patient's clinical profile is statistically closest to.^29^ This statistical approach calculates the risk of a new patient developing STDR within 1 year from the time of prediction, and this risk can be updated each time new data become available for the patient.

The transition from no DR to mild NPDR/BDR was modelled using a bivariate generalized linear mixed‐effects model that takes into account the correlation between measurements at different time points for the same patient. It is bivariate because it captures the measurements from both the right and left eye in a single model. The grading in each eye was considered as a binary longitudinal variable, with 0 representing no DR and 1 denoting mild NPDR/BDR. Correlation between repeated measurements for a patient and between retinopathy grading in each eye was modelled using random (patient‐specific) intercept terms in the mixed model. A two‐component mixture of Gaussian distributions was specified to allow flexible modelling of the joint distribution of random effects. Longitudinal models included clinical risk factors that influence the changes in retinopathy level over time. Because of the complexity of the statistical model, the Markov chain Monte Carlo method was used to estimate model parameters.^29^, ^30^

Penalized expected deviance alongside a forward selection approach, in combination with clinical judgment, was applied to identify the relevant demographic and clinical risk factors that influence changes in retinopathy level over time. Models were compared using penalized expected deviance, which penalizes for model complexity and is suitable for complex hierarchical models.^31^ As a result of the stochastic nature of the Markov chain Monte Carlo model, it is possible that different random starting values (seeds) generate slightly different models. Hence, in order to check the stability of the model, the process was generated for multiple seeds. Two training datasets, involving data from 70% of patients in each of the two prognostic groups, were used to build the model and data from the remaining 30% were used to test the predictive accuracy of the model. Training and test sets were randomly generated 100 times and the results were averaged. The statistical analyses were performed in R version 3.0.2 using the package mixAK.^32^

To allow for the fact that patients have been observed for different lengths of follow‐up period, time since first screening was included as a covariate in the longitudinal models. All patients had been followed up for a minimum of 2 years. To develop the models, we considered the period of time from the start of their observations up until the point 1 year before their final visit (in order to be able to predict the clinical status 1 year after). For patients who developed STDR, the final visit was defined as the time at which STDR was detected (ie, data beyond STDR detection were ignored). For patients who did not develop STDR the final visit was the last recorded visit; therefore, group memberships (whether or not the patient developed STDR within 1 year of the prediction visit) were known for all patients.

The fitted mixed models, one for each prognostic group, were used in a longitudinal discriminant analysis to predict the likelihood that a new patient would/would not develop STDR within 1 year. In particular, the likelihood of the new patient's data coming from each of the two mixed models was assessed and then weighted by the prevalence of each group to give a probability of developing STDR within 1 year. If this probability was greater than a threshold (chosen through analysis of a receiver‐operating characteristic [ROC] curve), then the patient was classified as developing STDR within 1 year and otherwise they were classified as non‐STDR.

The two prognostic models were subsequently used to predict for a new patient (test set) the likelihood of developing/not‐developing STDR within 1 year. Intuitively, the patient is linked to the group with the model the new patient's profile is closer to. The level of accuracy of the multivariate approach was assessed using the area under the ROC curve and its 95% confidence interval (CI). We also assessed the values of sensitivity (percentage of patients among those who truly developed STDR, who were correctly identified by the model), specificity (percentage of patients among those who did not develop STDR, who were correctly identified by the model) and the probability of correct classification (percentage of patients correctly classified), with their corresponding 95% CIs. The threshold chosen was associated with the point on the ROC curve nearest to the top left corner (ie, it provides the best balance in terms of number of patients correctly identified as not developing STDR and those correctly identified as developing STDR).

### 2.4 Simple stratification rules

Annual screening for DR has been adopted by several national screening programmes.^27^, ^28^, ^33^ Risk‐based stratified screening intervals are likely to be introduced in a number of countries within the next few years in order to cope with the imminent significant rise in the number of PWD. We explored with our data the overall accuracy of prediction using simple stratification rules based on retinopathy level alone to identify low‐ and high‐risk patients (the latter group consisting of patients who are likely to develop STDR within 1 year). Ideally, low‐risk patients could be offered 2‐year screening intervals or longer intervals. We defined sensitivity as the percentage of patients, out of the patients who developed STDR within 1 year, who are correctly predicted by the rule (and therefore allocated to annual screening intervals). Specificity was defined as the percentage of patients of those who did not develop STDR within 1 year, who were correctly predicted by the rule as not developing STDR within 1 year (and therefore were allocated to biennial screening intervals). We also calculated the reduction in the number of screening episodes achieved by simple stratification rules when compared with the currently recommended annual screening.

Subgroup analyses were conducted to further explore the effect of diabetes type on the classification performance.

## 3 RESULTS

The 7‐year data from 13 103 PWD were included in our model. The median follow‐up was 6 years between March 2009 and January 2016. The demographic and clinical characteristics by prognostic group are provided in Table 1. Screening visits for which model covariates were not available were excluded from the final model (12%). Compared with the non‐STDR group, we found that patients who developed STDR during the follow‐up period were more likely to be men, to be younger, to have type 1 diabetes, a longer disease duration and a higher HbA1c level, and were more likely to have missed screening appointments.

**Table 1:** Summary measures of demographic, clinical and level of retinopathy data

Variable	All patients	Non‐STDR group	STDR group
Number of patients	13 103	12 762	341
Total screening visits	49 520	48 562	958
Sex: female, n (%)	5469 (41.7)	5350 (41.9)	119 (34.9)
Age at first visit^a^, yrs	59.47 (13.34)	59.65 (13.27)	52.93 (14.43)
Type 1 diabetes, n (%)	651 (5.0)	585 (4.6)	66 (19.4)
Duration of diabetes^b^, yrs	1.95 (0.29, 4.73)	1.84 (0.28, 4.59)	4.95 (2.26, 9.28)
Follow‐up length^b^, yrs	6.18 (3.33, 8.84)	6.20 (3.32, 8.89)	5.51 (3.66, 7.30)
Ethnicity: white/non‐white/not reported, %	76/6/18	76/6/18	69/13/18
HbA1c^b^, mmol/mol	51 (44, 60)	50 (44, 59)	66 (53, 87)
HbA1c^b^, %	6.8% (6.2%,7.6%)	6.7% (6.2%,7.5%)	8.2% (7%,10.1%)
Cholesterol^a^, mmol/L	4.18 (1.01)	4.18 (1.01)	4.25 (1.07)
DBP^a^, mmHg	75.2 (9.15)	75.16 (9.15)	77.09 (9.18)
SBP^a^,mmHg	131.52 (14.08)	131.47 (14.06)	134.27 (15.04)
HDL cholesterol^a^, mmol/L	1.26 (0.37)	1.26 (0.37)	1.23 (0.41)
LDL cholesterol^a^, mmol/L	2.1 (0.85)	2.1 (0.85)	2.2 (0.84)
eGFR value^b^, mL/min/1.73 m^2^	76 (63, 88)	76 (63, 88)	84 (72, 90)
Missed appointment at previous visit before prediction, n (%)	631 (4.8%)	551 (4.3)	80 (23.5)
Retinopathy grades at first visit, %			
R0/R0	75	77	19
R0/R1	16	16	22
R1/R1	9	7	59
Retinopathy grades at prediction visit, %			
R0/R0	78	79	12
R0/R1	13	13	14
R1/R1	9	8	74

Abbreviations: BDR, background diabetic retinopathy; DBP, diastolic blood pressure; DR, diabetic retinopathy; HbA1c, glycated haemoglobin; NPDR, non‐proliferative diabetic retinopathy; SBP, systolic blood pressure; STDR, sight‐threatening diabetic retinopathy.

Retinopathy grades: R0 = non‐DR; R1 = mild NPDR/BDR.

a Values are mean (SD) and

b Median (interquartile range), reported as measures of location and variability.

As expected, the majority of patients who developed STDR (74%) within 1‐year screen interval exhibited mild NPDR/BDR in both eyes at their previous screen visit (prediction visit); only a small percentage of patients (12%) who developed STDR showed no DR in either eye at their previous screen visit. This trend was reversed for patients who did not develop STDR (79% showed no DR in both eyes and only 8% showed mild NPDR/BDR in both eyes at the time of prediction).

As expected, the risk factors for the progression from no DR to mild NPDR/BDR showed similar odds ratios (ORs) for the right and left eyes, with similar interpretation (data from both eyes were jointly modelled; the full model specification is given in Table S1). For simplicity, we report one OR for each risk factor. Ethnicity and estimated glomerular filtration rate (eGFR) were not included in the model because of the high rates of missing values observed (18% and 36%, respectively) and the lack of ethnic representation (predominantly white).

### 3.1 Multivariate model for patients who developed STDR

For patients who developed STDR within 1 year, the multivariate model showed that progression from no DR to mild NPDR/BDR during the follow‐up period prior to STDR was associated with diabetes duration with an OR (per 5 years of disease duration) of 1.78 (95% CI 1.21‐2.62) and with their previous screening appointment missing with an OR of 2.12 (95% CI 1.05‐4.42). Other factors such as sex, age, HbA1c, SBP and DBP, diabetes type and cholesterol level were not found to be statistically significant. The variable HbA1c was included in the final model for completion (despite its lack of significance in this group) because of its relevance as identified in the literature. The inclusion of HbA1c nonetheless did not affect the clinical interpretation of the other coefficients in the model.

### 3.2 Multivariate model for patients who did not develop STDR

For patients who did not develop STDR, progression from no DR to mild NPDR/BDR was associated with diabetes duration with an OR (per 5 years' disease duration) of 2.25 (95% CI 2.10‐2.40) and type 1 diabetes with an OR of 2.44 (95% CI 1.88‐3.21). Also, in this group the transition from no DR to mild NPDR/BDR was less likely to occur as time progressed (OR 0.97 per year; 95% CI 0.95‐0.99) as opposed to the STDR group, for whom the transition from no DR to mild NPDR/BDR was more likely to occur as time increased (OR 1.60 per year, 95% CI 1.33‐1.93). SBP and HbA1c were found to have statistically significant associations, although the ORs showed a lower effect compared with the risk factors listed above (for SBP every 10 mm Hg, OR 1.07 [95% CI 1.04‐1.10] and for HbA1c every 10 mmol/mol, OR 1.04 [95% CI 1.01‐1.07]).

The random intercepts of the model account for the within‐patient variation and encapsulate the underlying “state of health” of the patient not explained by the observable covariates. Within the group who developed STDR, there were two subgroups of patients: a group of just under a third of patients who had a high initial risk of progressing from no DR to mild NPDR/BDR (weight = 29.2% in Table S1) and a remaining group with a lower initial risk of progression (although in both cases patients' initial risk of progression was much higher than in the “no STDR” group). In the group of patients who did not develop STDR, just over a third of the patients belonged to a group with a very low initial risk of progression from no DR to mild NPDR/BDR (weight = 36.2%), with the remaining patients having a higher initial risk. The model takes into account the correlation between the right and left eye outcomes through the covariance matrices.

### 3.3 Accuracy of the multivariate discriminant tool

**Figure 1:**
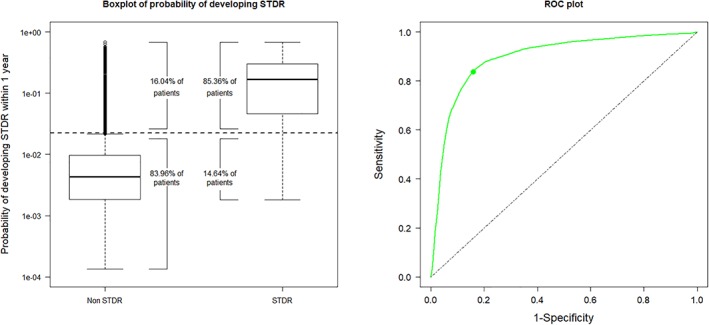
Boxplots for the predicted probability of developing sight‐threatening diabetic retinopathy (STDR) within 1 year when applying our multivariate discriminant model (left panel). The boxplots display the distribution of the predicted probabilities (1.5 × interquartile range below the first quartile, first quartile, median, third quartile and 1.5 × interquartile range above the third quartile). Values greater than 1.5 × interquartile range above the third quartile (or lower than 1.5 × interquartile range below the first quartile) are shown separately as plotted points (outliers). Receiver‐operating characteristic (ROC) curve (right panel) with sensitivity (85.4%) and specificity (84.0%) illustrated by the green dot

**Figure 2:**
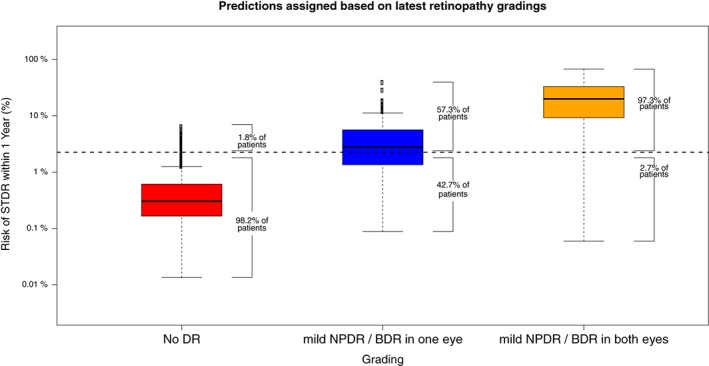
Boxplots for the predicted probability of developing sight‐threatening diabetic retinopathy (STDR) within 1 year for patients with no diabetic retinopathy (DR) at the time of prediction (left), mild non‐proliferative DR (NPDR)/background DR (BDR) in one eye (middle), and mild NPDR/BDR in both eyes (right)

The level of accuracy of the multivariate approach shown by Figure 1 indicates that 85.4% of patients who developed STDR within 1 year were correctly identified by the model (sensitivity, 95% CI 80.4‐89.7) and 84.0% of patients who did not were correctly identified by the model (specificity, 95% CI 80.7‐87.6). The area under the ROC curve (ROC AUC) was 0.90 (95% CI 0.86‐0.92). The probability of correct classification was 84.0% (95% CI 80.4‐89.7). Figure 2 shows the percentage allocated to each prognostic group based on the level of retinopathy at the time of prediction. Only 1.8% of patients who showed no DR at the time of prediction were predicted to develop STDR within 1 year (ie, the predicted risk was greater than the selected cut‐off for 1.8% of patients in this group). This percentage increased to 57.3% for patients who had mild NPDR/BDR in one eye only, and to 97.3% for patients who had mild NPDR/BDR in both eyes at the time of prediction.

The effect of diabetes type on the classification performance was further explored by conducting subgroup analyses. For patients with type 1 diabetes, our model achieved an ROC AUC of 0.81, with optimal sensitivity of 84.3% and 78.0% specificity. For patients with Type 2 diabetes, the ROC AUC was 0.90, with optimal sensitivity of 85.6% and specificity of 84.3%.

### 3.4 Comparison with simple stratification rules

**Figure 3:**
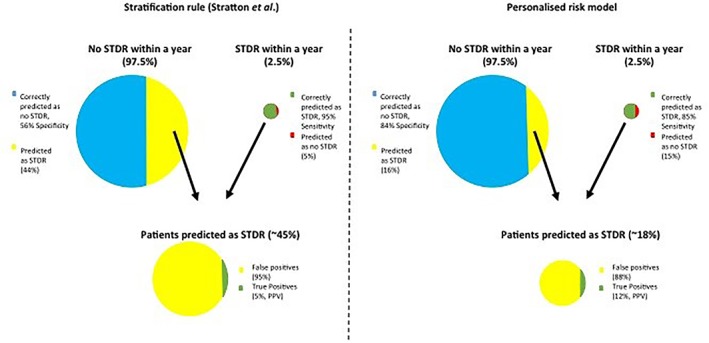
Prediction accuracy of the two‐episode stratification rule versus our personalized risk model

When we assessed the overall predictive accuracy of the simple stratification rules to identify low‐ and high‐risk patients, we observed that if we applied the simple rule that patients with no retinopathy in either eye during a 2‐year period with two successive annual screening episodes are predicted as not developing STDR within 1 year, and otherwise as developing STDR,^25^, ^26^ the sensitivity and specificity values were 95.2% (95% CI 92.2‐97.6) and 55.7% (95% CI 54.5‐57.0), respectively (Figure 3, left panel). Alternatively, if only the level of retinopathy of the screening episode at the time of prediction was considered, and patients were predicted as not developing STDR if neither of the eyes showed DR, and as developing STDR otherwise,^27^ the sensitivity and specificity values were 87.5% (95% CI 82.4‐92.7) and 79.5% (95% CI 78.6‐80.4), respectively (Table S2). While the sensitivity dropped by ~8% with the second rule compared with the first rule, the specificity increased by 24%, and consequently we observed a significant reduction in the number of screening episodes required (from a reduction of 27% with the first rule to a reduction of 39% with the second rule). The probabilities of correct classification were 56.7% (95% CI 55.5‐58.0) and 79.7% (95% CI 78.8‐80.6) for the first and second stratification rules, respectively.

Statistical comparisons in sensitivity and specificity between the multivariate approach and the two simple stratification rules demonstrated that, while the level of sensitivity obtained using the first rule was significantly higher when compared to the multivariate approach (9.8% difference, [95% CI 4.9,14.7]), the specificity of the multivariate approach was significantly higher when compared with the first rule (28.3% difference [95% CI 24.9‐32.3]). The multivariate approach also showed a significantly higher level of specificity when compared with the second rule (4.5% difference [95% CI 0.8‐8.2]), while the increment in sensitivity by the second rule was not statistically significant at the 95% confidence level (2.1% difference (95% CI −0.2‐7.4]).

## 4 DISCUSSION

We are heading towards personalized medicine, whereby patient management can be tailored based on individual risk of disease or response to treatment. In particular, the predicted risk of developing STDR can be used to recommend personalized screening intervals. Annual screening is currently adopted in many national screening programmes at a considerable cost to health services.^9^ Longer screening intervals for those at low risk of developing STDR have already been introduced, for example, in Iceland, using a model that accounts for the level of retinopathy as well as other clinical information, including type and duration of diabetes, HbA1c level and blood pressure.^34^, ^35^, ^36^ Additional models have recently been proposed in the literature to tailor screening intervals for DR.^8^, ^37^ Longer screening intervals for patients with a low risk of developing STDR are expected to be implemented in England and Wales within the next few years to cope with the increased economic burden triggered by the steady increase in disease prevalence and the lack of extra funding for screening. Risk‐based approaches are timely in that they could be used to identify patients at high risk so that they can be closely monitored and treated earlier, while the majority of patients at low risk can be screened less often, allowing the optimization of limited health resources.

In the present paper, we report the results of a recently developed multivariate longitudinal approach^29^, ^30^ to predict the risk that a given patient with diabetes will develop STDR within 1 year. The model shows high levels of classification accuracy (sensitivity and specificity were 85.4% and 84.0%, respectively). The AUC was 0.90, which is higher than the AUCs previously reported in Scanlon et al.^9^ and Aspelund et al.^35^ of 0.79 and 0.76, respectively. A similar AUC (0.90) was reported in Eleuteri et al.^8^ and although the specificity reported by these authors was 90%, the level of sensitivity was much lower than the sensitivity achieved with our multivariate model (67% vs. 85.4%).

There are a number of advantages to our approach. From a methodological point of view, the approach is robust against misspecification of the distribution of the random effects term, which is a term that takes into account the correlation between measurements at different time points in the model.^29^ The approach has the potential to develop dynamic models with which the risk can be recalculated every time new data from the patient become available.^29^, ^30^ All 92 practices approached within the Liverpool area agreed to participate, which demonstrates the screening coverage data.

Limitations of this study include the possible misclassification in level of retinopathy during grading, the fact that the costs of misclassification were not considered (which differ between STDR and non‐STDR misclassification) and the fact that only internal validation was conducted. We acknowledge that a comprehensive assessment of the model's predictive performance would require external validation using data from a different cohort; for example, the predictive accuracy of our model needs to be validated for different ethnic groups. Our dataset, which included predominantly white patients, did not include representative samples from different ethnic groups. The differences in performance between our approach and the stratification rules must be explored using different cohorts to confirm the reproducibility of our findings.

Several multivariate regression models have been proposed over the years to predict DR risk.^9^, ^15^, ^16^, ^35^, ^36^, ^38^ In these models, retinopathy grading and clinical variables are treated as predictor variables in the model. For example, Aspelund et al.^35^ proposed a risk algorithm for development of STDR based on the Weibull proportional hazard model. The predicted tool included type and duration of diabetes, HbA1c, blood pressure and presence of NPDR (defined as a binary variable: Yes/No). The recommended screening intervals generated by their risk model (which ranged from 6 months to 5 years) were estimated to achieve 59% fewer visits than with annual screening. This reduction was larger than the reduction achieved with our predictive model (41% reduction) because screening intervals >2 years were considered in their allocation; however, the accuracy achieved by their model was lower (eg, 0.76 vs. 0.90 for the AUC) and a significant limitation of their model is that it uses historical data on risk factors. Model coefficients in Aspelund et al.^35^ were fitted separately for type 1 and type 2 diabetes, while type of diabetes was added here as a covariate in the longitudinal models. The subgroup analysis conducted by type of diabetes indicates that our classification tool is comparably sensitive, irrespective of type of diabetes, with a small reduction in specificity in the patients with the less common Type 1. We believe this provides confidence that the model can classify well regardless of the type of diabetes.

The concept behind our approach is different from previously proposed multivariate regression models. The profiles of level of retinopathy over time and prior to STDR development are first modelled using demographic and primary care data, and the risk that a new patient at a specific time point will develop STDR is estimated based on these profiles (which are in turn driven by demographic and time‐dependent primary care data). Markov chain models are based on a similar idea, where the risk for moving from one state to another state is modelled, and where state is defined by level of retinopathy.^8^, ^37^, ^39^ Rather than proposing an alternative predictive model to predict STDR in competition with those already developed, we devised an alternative model, with similar levels of accuracy that naturally describes the influence of clinical data (primary care/missing appointments data) on progression of DR and how these relationships affect the risk of developing STDR.

The models discussed above focus on DR progression. The identification of DR in newly diagnosed individuals has been also subject of research. For example, in Cichosz et al.,^40^ a linear classification tool was proposed to predict mild or moderate NPDR in people with newly diagnosed type 2 diabetes.

We acknowledge that alternative techniques, such as neural networks, could also be considered. Despite their advantages, such as an ability to detect non‐linear relationships between the outcome and dependent variables, their limitations include the inability to form a clinical interpretation (ie, seen as a “black box”) and tendency to overfitting. The advantage of the method we have proposed is that it can deal with the complex structure of the data (ie, longitudinal data collected over time, correlation between the measures from the right and left eye, outcome variables of different type) while allowing the clinical interpretation of the model.

In territories with established screening programmes, a clinical debate is taking place on whether known clinical factors for development and progression of DR (such as duration, HbA1c and type of diabetes) should be used in a decision model to tailor screening intervals for DR. Given that retinopathy level is the most informative factor with regard to progression to STDR, a simple rule that makes use of this biomarker alone is being considered to determine risk‐based DR screening intervals in the United Kingdom.^26^ The English Diabetic Eye Screening Programme has proposed an extension to 2‐year intervals for patients with no retinopathy in either eye during a 2‐year period with two successive annual episodes. Our data suggest that if we apply this simple rule, for which patients with no retinopathy in either eye during a 2‐year period with two successive annual screening episodes are recommended biannual screening intervals, and annual screenings otherwise, a high level of sensitivity would be achieved (95%). This means that only 5% of patients who develop STDR within 1 year would be allocated to a 2‐year screening interval. A key limitation of such a rule becomes evident if the reduction in the number of eye examinations (compared with annual screening) is not sufficient to cope with the steady increase in the number of PWD, given the lack of extra funding for screening. In particular, there are a number of limitations associated with this rule. These are as follows. (a) Our data suggest that this rule lacks accuracy to identify low‐risk patients (56% specificity; Figure 3, left panel) and 44% of patients who will not develop STDR would be invited to annual screening, which affects a considerable number of patients given the low incidence rate of STDR. Multivariate risk‐based models are likely to offer a more cost‐effective solution. The statistical risk model in Aspelund et al.^35^ and the one proposed in the present study could generate a reduction in the number of screening episodes of >40%, while achieving acceptable values of sensitivity. (b) The acceptability to patients and staff is an important aspect that has not yet been evaluated. The acceptability of variable interval screening in a pragmatic whole population based on the impact on attendance rates to screening should be taken into account (this consideration applies to any model or rule considered for implementation). As two successive annual screening episodes are required for stratification, patients with one of the screening episodes missing would have to be allocated to the high‐risk group, making the allocation less efficient.

A simpler stratification rule is the one that considers the level of retinopathy of the screening episode at the time of prediction only. Despite its simplicity, it is effective in reducing the number of screening episodes (showing a reduction of 39% compared to a reduction of 27% with the first rule) at the cost of a drop in sensitivity by 7% (88% sensitivity). The recommendation on screening for referable retinopathy by the Scottish Intercollegiate Network (updated version from 2014)^27^ is based on this second simple rule (although the first rule is expected to be soon introduced in Scotland).

Figure 3 compares the accuracy of the two‐episode stratification rule and our multivariate approach. While the two‐episode stratification rule identified more patients who developed STDR within a 1‐year screen interval when compared to the multivariate risk model (95% vs. 85%), it also identified fewer patients who did not develop STDR (56% vs. 84%). Given the low annual incidence rate of STDR (~2.5%), the low specificity of the two‐episode stratification rule would lead to a very low positive predictive value (5%). In other words, the majority of patients classified as developing STDR within 1 year would not develop STDR (false‐positives, yellow areas in Figure 3). With the multivariate risk model the positive predictive value doubles.

We jointly modelled clinical data and retinopathy to predict STDR accurately. A substantial body of evidence suggests that changes in the values of certain risk factors has a beneficial effect on outcomes in diabetic retinal diseases.^3^, ^27^, ^41^, ^42^ The multivariate predictive model we have developed uses baseline clinical data to model changes in DR (transitions among the states no DR and mild NPDR/BDR in either eye). We conclude that long‐term progression of DR is driven by the patient's overall clinical profile with respect to diabetes control and that a risk prediction model using systemic risk factor data, as well as retinopathy level, may offer a better trade‐off between achieving an acceptable sensitivity, while also keeping a desirable specificity.

## Supporting information


**Figure S1.** Schematic diagram illustrating the classification process. The levels of DR in both eyes were jointly modeled using both baseline and time‐dependent information until the time of prediction using the training dataset (left panel). Two multivariate models were generated: one for the STDR group and one for the non‐STDR group. A discriminant model was generated using the parameters derived from the two multivariate longitudinal models (one for each prognostic group). The risk that a new patient would develop STDR within 1 year was estimated using the longitudinal clinical data of the new patient.

**Table S1.** Estimates of the model coefficients of the two bivariate generalized linear mixed‐effects models (one for each prognostic group) where the outcome (binary) is progression from no DR to mild non‐proliferative DR / background DR (mild NPDR / BDR). The odds ratios and corresponding 95% confidence intervals are provided.

**Table S2.** Predictive accuracy of our multivariate approach and of the simple stratification rules to identify patients who will/will not develop STDR within a one‐year screen interval.
