# Supplementary material for: Personalized risk‐based screening for diabetic retinopathy: A multivariate approach versus the use of stratification rules
Source: Diabetes Obes Metab. 2018 Oct 30;21(3):560–8. doi: 10.1111/dom.13552 (PMC6492102; doi:10.1111/dom.13552)
Supplement: Supplementary file 1 — Figure S1. Schematic diagram illustrating the classification process. The levels of DR in both eyes were jointly modeled using both baseline and time‐dependent information until the time of prediction using the training dataset (left panel). Two multivariate models were generated: one for the STDR group and one for the non‐STDR group. A discriminant model was generated using the parameters derived from the two multivariate longitudinal models (one for each prognostic group). The risk that a new patient would develop STDR within 1 year was estimated using the longitudinal clinical data of the new patient. [file DOM-21-560-s001.docx]

Figure 1. Schematic diagram illustrating the classification process. The levels of DR in both eyes were jointly modeled using both baseline and time-dependent information until the time of prediction using the training dataset (left panel). Two multivariate models were generated: one for the STDR group and one for the non-STDR group. A discriminant model was generated using the parameters derived from the two multivariate longitudinal models (one for each prognostic group). The risk that a new patient would develop STDR within one year was estimated using the longitudinal clinical data of the new patient.
