# Supplementary material for: Personalized risk‐based screening for diabetic retinopathy: A multivariate approach versus the use of stratification rules
Source: Diabetes Obes Metab. 2018 Oct 30;21(3):560–8. doi: 10.1111/dom.13552 (PMC6492102; doi:10.1111/dom.13552)
Supplement: Supplementary file 2 — Table S1. Estimates of the model coefficients of the two bivariate generalized linear mixed‐effects models (one for each prognostic group) where the outcome (binary) is progression from no DR to mild non‐proliferative DR / background DR (mild NPDR / BDR). The odds ratios and corresponding 95% confidence intervals are provided. [file DOM-21-560-s002.docx]

| **STDR group** | | | | | | | | |
| --- | --- | --- | --- | --- | --- | --- | --- | --- |
| **Model coefficients** |  | **Odds ratio** | **95% lower** | **95% upper** |  | **Odds ratio** | **95% lower** | **95% upper** |
| Time (per year) | **Left eye** | 1.626 | 1.349 | 1.980 | **Right eye** | 1.590 | 1.340 | 1.911 |
| Duration (per 5 years) |  | 1.782 | 1.212 | 2.617 |  | 1.854 | 1.295 | 2.741 |
| HbA1c (per 10mmol/mol) |  | 0.985 | 0.870 | 1.137 |  | 1.086 | 0.953 | 1.237 |
| Missing (binary) |  | 2.123 | 1.052 | 4.420 |  | 3.320 | 1.651 | 7.495 |
| **First Mixture Component** |  | **Estimate** | **95% lower** | **95% upper** |  | **Estimate** | **95% lower** | **95% upper** |
| Weight |  | 0.708 | 0.565 | 0.975 |  | 0.708 | 0.565 | 0.975 |
| Mean (random intercept) |  | -0.373 | -1.754 | 1.319 |  | -1.113 | -2.429 | 0.588 |
| SD (random intercept) |  | 4.736 | 2.268 | 9.622 |  | 4.433 | 1.712 | 8.240 |
| Covariance(Left Eye Intercept :Right Eye Intercept) |  | 3.642 | 2.028 | 9.322 |  | 3.642 | 2.028 | 9.322 |
| **Second Mixture Component** |  | **Estimate** | **95% lower** | **95% upper** |  | **Estimate** | **95% lower** | **95% upper** |
| Weight |  | 0.292 | 0.025 | 0.435 |  | 0.292 | 0.025 | 0.435 |
| Mean (random intercept) |  | 15.519 | -3.464 | 32.374 |  | 13.780 | 2.809 | 36.199 |
| SD (random intercept) |  | 6.367 | 0.152 | 38.248 |  | 7.552 | 0.149 | 48.568 |
| Covariance(Left Eye Intercept:Right Eye Intercept) |  | 0.795 | -11.889 | 18.792 |  | 0.795 | -11.889 | 18.792 |

| **No STDR group** | | | | | | | | |
| --- | --- | --- | --- | --- | --- | --- | --- | --- |
| **Model coefficients** |  | **Odds ratio** | **95% lower** | **95% upper** |  | **Odds ratio** | **95% lower** | **95% upper** |
| Time (per year) | **Left eye** | 0.971 | 0.952 | 0.991 | **Right eye** | 0.985 | 0.965 | 1.005 |
| Duration (per 5 years) |  | 2.247 | 2.101 | 2.402 |  | 2.212 | 2.071 | 2.368 |
| Diabetes Type (1=Type 1) |  | 2.443 | 1.884 | 3.209 |  | 2.775 | 2.122 | 3.670 |
| HbA1c (per 10mmol/mol) |  | 1.038 | 1.009 | 1.067 |  | 1.029 | 1.000 | 1.058 |
| SBP (per10 mm/Hg) |  | 1.068 | 1.040 | 1.097 |  | 1.051 | 1.021 | 1.081 |
| **First Mixture Component** |  | **Estimate** | **95% lower** | **95% upper** |  | **Estimate** | **95% lower** | **95% upper** |
| Weight |  | 0.362 | 0.242 | 0.481 |  | 0.362 | 0.242 | 0.481 |
| Mean (random intercept) |  | -5.701 | -6.684 | -4.949 |  | -5.291 | -6.097 | -4.528 |
| SD (random intercept) |  | 17.714 | 12.975 | 25.320 |  | 16.085 | 12.088 | 21.893 |
| Covariance(Left Eye Intercept :Right Eye Intercept) |  | 13.560 | 10.437 | 18.638 |  | 13.560 | 10.437 | 18.638 |
| **Second Mixture Component** |  | **Estimate** | **95% lower** | **95% upper** |  | **Estimate** | **95% lower** | **95% upper** |
| Weight |  | 0.638 | 0.519 | 0.758 |  | 0.638 | 0.519 | 0.758 |
| Mean (random intercept) |  | -4.320 | -4.783 | -3.890 |  | -4.161 | -4.630 | -3.728 |
| SD (random intercept) |  | 2.167 | 1.521 | 2.843 |  | 2.544 | 1.883 | 3.265 |
| Covariance(Left Eye Intercept:Right Eye Intercept) |  | 2.124 | 1.578 | 2.675 |  | 2.124 | 1.578 | 2.675 |

Table 1. Estimates of the model coefficients of the two bivariate generalized linear mixed-effects models (one for each prognostic group) where the outcome (binary) is progression from no DR to mild non-proliferative DR / background DR (mild NPDR / BDR). The odds ratios and corresponding 95% confidence intervals are provided.
