# Supplementary material for: Personalized risk‐based screening for diabetic retinopathy: A multivariate approach versus the use of stratification rules
Source: Diabetes Obes Metab. 2018 Oct 30;21(3):560–8. doi: 10.1111/dom.13552 (PMC6492102; doi:10.1111/dom.13552)
Supplement: Supplementary file 3 — Table S2. Predictive accuracy of our multivariate approach and of the simple stratification rules to identify patients who will/will not develop STDR within a one‐year screen interval. [file DOM-21-560-s003.docx]

| **Multivariate discriminant model** | **Use of primary care data** | **Previous retinopathy profile** | **Retinopathy levels at time of prediction** | **Allocation rule** | **Overall accuracy** | **Reduction in the number of eye examinations (compared to annual screening)** |
| --- | --- | --- | --- | --- | --- | --- |
|  | Yes | Taken into account | Taken into account | 12 months screen interval if predicted risk >= threshold | **84.0%**  85.4% sensitivity  84.0% specificity | 41% |
|  |  |  |  | 24 months screen interval if predicted risk < threshold |  |  |
| **Stratton *et al.* rule**  **Two-episode stratification rule** | No | Previous screening episode  (1 year earlier) |  |  | **56.7%**  95.2% sensitivity  55.7% specificity | 27% |
|  |  | No retinopathy in either eye | No retinopathy in either eye |  |  |  |
|  |  |  |  | 24 months screen interval |  |  |
|  |  | No retinopathy in either eye | Retinopathy in one or both eyes | 12 months screen interval |  |  |
|  |  | Retinopathy in one or both eyes | No retinopathy in either eye |  |  |  |
|  |  | Retinopathy in one or both eyes | Retinopathy in one or both eyes |  |  |  |
|  |  | NA | Any |  |  |  |
| **One-episode stratification rule** | No | Not taken into account | No retinopathy in either eye | 24 months screen interval | **79.7%**  87.5% sensitivity  79.5% specificity | 39% |
|  |  |  | Retinopathy in one or both eyes | 12 months screen interval |  |  |

Table 2. Predictive accuracy of our multivariate approach and of the simple stratification rules to identify patients who will/will not develop STDR within a one-year screen interval
